# Daily life participation in PTSD: pilot study on patterns and correlators

**DOI:** 10.3389/fpsyt.2024.1429647

**Published:** 2024-07-25

**Authors:** Ruth Shapira, Yisca Jessica Baris Ginat, Lena Lipskaya-Velikovsky

**Affiliations:** ^1^ Day care ward, School of Occupational Therapy, Faculty of Medicine, the Hebrew University, Jerusalem, Israel; ^2^ The Jerusalem Mental Health Center, Jerusalem, Israel

**Keywords:** objective participation, subjective participation, neurocognition, functional capacity, environmental impacts

## Abstract

**Introduction:**

Participation in daily life activities with both the personal and community meaning is an important component of health and well-being. Even though there are mounting reports on the challenges in various aspects of daily-life functioning among individuals with post-traumatic stress disorder (PTSD), to date little research has been conducted on their comprehensive patterns of participation. The study aimed to describe objective and subjective participation dimensions in PTSD compared to healthy controls and investigate the association between personal and environmental factors and participation.

**Methods:**

Sixty-one individuals were enrolled in two groups: PTSD (*N*=31; age: *M*=34.3; women:77.4%) and healthy controls matched by age and gender. The PTSD group completed standard assessments for symptom severity, general cognition, executive function (EF), sensory processing, self-efficacy, functional capacity, and environmental properties. Both groups completed a participation questionnaire.

**Results:**

Individuals with PTSD participated with low intensity and diversity, more occupations were abandoned (-4.73<t<5.73, *p*<0.05), and less meaning was found in the included occupations. Participation objective dimensions were associated with self-reported EF (-0.47<r<-0.42, *p*<0.05), sensory modulation profile (2.51<t<2.81, p<0.05), and environmental properties (-0.44<r<0.5, *p*<0.05), but not with PTSD symptom severity, and objective measures of cognition and functional capacity. No correlators were identified for subjective participation dimensions.

**Discussion:**

The study demonstrates profound restrictions in participation in PTSD raising serious concerns. There are unique patterns of association between objective participation dimensions, subjective cognitive indices, sensory modulation, and environmental factors, suggesting a need for PTSD feature-specific intervention approaches to advance the participation of those with PTSD as a means of promoting health and well-being.

## 1 Introduction

People with post-traumatic stress disorder (PTSD) of any origin experience a range of symptoms that significantly impact their daily lives, including restrictions in everyday functioning. Despite evidence of functional challenges ensuing from PTSD, there is a lack of comprehensive understanding of the wide occupational landscape in various areas and dimensions. Additionally, research on the factors that influence everyday functioning in PTSD is limited. In contemporary discourse, both objective and subjective dimensions of everyday functioning across a range of occupations are recognized as pivotal elements of health (1). Therefore, augmenting our comprehension of this issue in PTSD is essential, as it holds the potential to significantly contribute to the health, well-being, and recovery of this population.

PTSD is characterized by the intrusive re-experience of a traumatic event, dissociative reactions, psychological distress at exposure to internal or external cues that symbolize or resemble an aspect of the traumatic event, negative alterations in cognitions, mood, arousal, and reactivity associated with the traumatic event, and avoidance (DSM-5; ICD-11) (2, 3). Prevalence rates for PTSD vary widely around the world (4), but Israel has one of the highest rates, 9% (5), these, in addition to a 2.6% prevalence of complex post-traumatic stress disorder (CPTSD) (5), which is evoked by repeated exposure to traumatic event(s) and/or of multiple forms (ICD-11) (3). With the impact on social, vocational, and other important areas of functioning, PTSD has, unfortunately, a low treatment rate (6).

### 1.1 Participation of those with PTSD

The World Health Organization (WHO) places everyday functioning in the context of participation―a multi-dimensional concept representing the involvement of people in all areas of life (1). There is evidence of disruption in participation including self-care, productivity, leisure, and social participation in PTSD (7, 8). Within the scope of self-care activities, sleep disorders consistently were addressed (8). In addition, there is evidence of the difficulties in managing and maintaining health (9). Among productive activities, complications with obtaining and maintaining employment most commonly were reported (8, 10), however, community mobility, safe driving, financial management, meal planning, school functioning, and parenting all were also found to be interrupted (8). Involvement in leisure activities of cultural entertainment (e.g., cinema), outdoor recreation, sports, and social activities was found to be reduced following PTSD (8, 11). Additionally, people diagnosed with PTSD experienced difficulty in creating and maintaining meaningful relationships within the community and with their spouses (12–14). Despite mounting evidence on restrictions in general indices of functioning or participation in specific areas of occupations, to the best of our knowledge, little research was done on the broad participation patterns of PTSD. Indeed, each area of participation brings its unique contribution to daily life, well-being, and health which are worthy of attention. For example, employment brings financial stability, social status, opportunities for social networking, health management, and skills retention and development (15). Leisure activities provide opportunities to find personal strengths and abilities, generate positive emotions, help minimize the impact of symptoms, find meaning in everyday life, and establish companionship and meaningful relationships (16, 17). Still, healthy participation patterns are characterized by ongoing intervening between occupations of different areas, which is particularly relevant for mental health (15). In addition, participation patterns include subjective aspects of involvement of the experience of involvement (18) and are of critical importance for health and well-being. For example, people with PTSD reported a lack of interest and decreased satisfaction with work and parenting (19, 20), and, in the case of leisure, a disconnection between the activities defined as leisure and the individual experience of leisure (21), all of which affected health and well-being. Even though issues of experience within participation were evoked through various studies (e.g., 16, 17), they have scarcely been a target for investigation.

### 1.2 Participation enablers for those with PTSD

Participation in daily life activities results from the dynamic interplay among personal cognitive, affective, and physical factors, environmental characteristics, and occupational features (22). However, the investigation into the interplay among these components concerning participation has been relatively limited in PTSD, even though evidence supports this notion (23). Previous research indicates the impact of the PTSD symptoms’ severity and number on general indices of everyday functioning (24), vocational outcomes, parenting (25), and subjective and objective academic achievements (26), accounting for up to 42.9% of the variance (27). Specifically, symptoms that were found to be of relevance to the participation aspects are avoidance, emotional numbing, low motivation, stress, feelings of guilt, and fluctuations in arousal (28–30).

Coping with complications associated with PTSD necessitates resilience, which refers to the capacity to effectively confront significant adversity and adapt to it (31). Within the construct of resilience, self-efficacy―an individual’s confidence in their ability to attain a desired goal with the help of available resources (32)―holds considerable significance (33). Self-efficacy emphasizes positive expectations and personal agency in overcoming obstacles, focusing on beliefs rather than actual actions (33). Still, due to its pivotal role in goal-directed activities, self-efficacy has been associated with actual functioning in serious mental health conditions such as schizophrenia (34) and depression (35). Research among trauma survivors has demonstrated a direct association between self-efficacy and PTSD symptom severity (36, 37), but its relationship with participation has not been extensively explored.

There is robust evidence of objective neurocognitive deficits in PTSD, primarily in executive functions, sustained attention, working memory, learning, and information processing speed (23, 38, 39, 61). These are in addition to self-reporting on cognitive impairments (23). Although there is limited research in the field, the existing findings reveal a relationship between cognition and general indices of occupational and social achievements (23, 39, 40). Functional capacity represents the ability to perform everyday living skills in a controlled environment, was previously reported to be a valuable predictor of participation, and was found to be altered in PTSD (30, 41, 42, 61).

Sensory modulation is defined as a neural process involved in the registering and interpreting of sensory stimuli from various modalities to generate appropriate behavioral and emotional responses based on context and individual needs (43, 44). A range of sensory modulation aberrations has been observed in PTSD, all of which share a common feature: a low threshold for sensory stimuli and interference with the inhibition process (45–47). These sensory alterations―sensory modulation disorder―have been found to correlate with the severity of PTSD symptoms (45, 47). However, to date, the association between SMD and functional outcomes in individuals with PTSD has not been thoroughly investigated.

Participation in daily life activities is contingent upon the attributes of the physical, social, cultural, and institutional environment in which individuals reside, and where occupations take place (1, 22), in addition to personal factors. From this standpoint, a thorough examination of participation should consider the environment as a determining factor since what facilitates one individual or occupation might present obstacles for another.

### 1.3 Study aims

In summary, limited research has explored the comprehensive construct of participation and enabling factors in PTSD (23). Given the importance of participation in occupations for health, well-being, and recovery in PTSD, this pilot study’s aim was twofold: (1) to delineate the objective and subjective dimensions of participation across a range of areas in comparison to health controls; and (2) to comprehensively investigate personal and environmental factors that impede participation among individuals with PTSD of different source in Israel. This study may enhance understanding of potential mechanisms through which PTSD leads to restrictions in different dimensions of participation, and, may provide insights to guide the development of interventions aimed at promoting participation, health, and well-being.

## 2 Methods

This is a pilot cross-sectional and comparative study with a convenience sample of individuals with PTSD, matched by gender and age-healthy controls.

### 2.1 Participants

Sixty-one individuals participated in this study in two groups: those with PTSD (N=31) and healthy controls (N=30). Inclusion criteria for the PTSD group included (a) formal diagnosis of PTSD according to DSM-5 (2) and (b) men and women in the age range of 18–65. Exclusion criteria were (a) diagnosis of psychosis or mania; (b) current substance abuse; (c) significant neurological, physical, or developmental diagnosis that affects daily functioning; and (d) legal guardian. The control group (healthy controls – HC) included healthy volunteers recruited through convenience sampling from social networks. They were matched to the study group by gender and age. The inclusion criteria for this group were: (a) no history of neurological, neuropsychiatric, or motor health conditions according to self-report, and (b) no constant medication of any type, including analgetic. Those who were reported on neuroleptic medication in the past were excluded from the study.

The sample size was calculated based on known data from the literature on the relationship between functional capacity and a general cognitive score in PTSD (42). Given alpha = 0.05 and power of 0.85, N=29 was found (r = 0.48) (GPower software).

### 2.2 Measurements

#### 2.2.1 Participation and functional capacity

The Adults Subjective Assessment of Participation (ASAP; 48) questionnaire was used to assess objective and subjective participation dimensions in 52 activities, by the following areas of occupation (categories): (1) domestic life; (2) entertainment and recreation; (3) care for children and other adults; (4) learning and applying knowledge; (5) sport and physical activity; (6) self-care; (7) quiet leisure; and (8) vocation. The participant is asked to rate for each activity the following objective dimensions: number of participated activities (diversity; 0–52); intensity/frequency (0–9), where (at home/outdoors); with whom (alone/with others); and subjective dimensions of enjoyment (1–6) and satisfaction (1–6). Final scores are calculated for each area of occupation and the whole questionnaire. The ASAP has satisfactory test-retest reliability (0.553<r<1) and construct validity that was established through a factor analysis (2.12 <eigenvalue<5.79). Discriminant validity was established by demonstrating differences between people with different disabilities and healthy controls (3.12<F<7.67) (48). In addition, we added the scale on the participation meaning based on the Meaningful Activity Participation Assessment (MAPA, 49). The scale was rated on the 6-point Likert scale (0-not meaningful; 5- most meaningful) with total score calculation like those of the MAPA.

USCD Performance-based Skills Assessment (UPSA; 50) was applied to evaluate functional capacity in five areas: medication management, financial management, using the telephone and communication, using public transportation, and planning leisure activities. This performance-based test comprises a simulation of 11 daily life tasks. The final score ranges from 0–100. The UPSA has acceptable test-retest reliability (r=0.74), criterion validity (r=0.86), convergent validity with cognitive tests (r=0.60 to r=0.79), and discriminant validity between different groups of mental diagnosis and healthy controls.

#### 2.2.2 Personal factors

PTSD Checklist for DSM-5 (PCL-5; 51) is a self-report questionnaire for adults to evaluate the presence of PTSD symptoms, according to the 5DSM criteria. The questionnaire includes 20 items. For each item, the subject is asked to describe how much the described problem bothered him during the last month, on a 5-point Likert scale (0=not at all, 4=extremely). There is a final score of all items and subscale scores, that represent clusters of the PTSD symptoms: (a) intrusive thinking; (b) withdrawal symptoms; (c) changes in cognitive functions and mood; and (d) overstimulation. The cut-off score of 33 is used for the diagnosis of PTSD. Following psychometric properties were established for the tool: internal consistency (Cronbach α = 0.96) and test-retest reliability (r = 0.85). Construct, discriminant, and convergent validity are established as well.

Pittsburgh Sleep Quality Index (PSQI; 52) was used to assess sleep quality and disturbances over a one-month time interval based on self-report. The questionnaires address subjective sleep quality, sleep latency, sleep duration, habitual sleep efficiency, sleep disturbances, and use of sleeping medication. The sum of scores for the components yields one global score. The questionnaire has good internal consistency (Cronbach α=.83) and discriminating validity between a population with sleep disorders and a non-clinical population (53).

The General Self-Efficacy Scale (GSFS; 54) is a self-reporting questionnaire that assesses a person’s general sense of mastery and capability. The questionnaire contains 14 items rated on a five-point Likert scale (1 = not at all, 5 = very much). The total score of the GSE has sufficient internal consistency (a=0.87-0.95) and concurrent validity (0.88-0.98).

Montreal Cognitive Assessment (MoCA; 55) was used for the evaluation of general cognitive functioning. The tool includes 30 items divided into 7 cognitive domains: (1) attention and concentration; (2) executive functions; (3) immediate memory; (4) language; (5) abstraction; (6) delayed memory; and (7) orientation. A sum score of 26 or higher is considered normal cognitive functioning. The MoCA demonstrates good internal consistency (α=0.83), criterion validity with additional cognitive tests, and discriminate validity indicating individuals with mild cognitive disorders (94.6%).

Trail Making Test, Parts A & B (TMT; 56) is a widely used paper-and-pencil test for speed of processing and mental flexibility. The completion time for each part was registered. There is a well-established test-retest reliability, internal consistency between the two parts, and concurrent validity.

Dysexecutive Questionnaire Self-reporting (DEX-S; 57) is a self-reporting questionnaire for adults to assess daily problems related to executive function impairment. DEX-S includes 20 items organized into 4 domains: emotion/personality, motivation, behavior, and cognition. The items are rated on a five-point Likert scale (0=never, 4=very often). The total score and domains’ sub-score may be calculated for the instrument. DEX-S has internal consistency with an alpha coefficient of 0.85 and convergent validity.

The Adolescent/Adult Sensory Profile (AASP; 58) is a self-reporting questionnaire for adolescents and adults to assess impaired sensory processing patterns, based on Dunn (58) sensory processing model. The questionnaire includes 60 statements concerning each of the sensing systems, as represented in daily life situations. The individual reaction on how often there is a reaction to the described sensory event is scored with a five-point Likert scale (1=almost never, 5=almost always). The sub-scores address four sensory patterns based on normative data: (a) low registration; (b) sensory seeking; (c) sensory sensitivity; and (d) sensory avoidance. The questionnaire has well-established psychometric properties including internal consistency (0.639<Cronbach α<0.699), discriminant and convergent validity. In this study, due to the small sample, we classify the sensory processing patterns into two groups: typical―similar to the average, or atypical―different from the average.

#### 2.2.3 Environment

The Occupational Self-Assessment (OSA; 59), environment sub-scale was used to address individual perception of the environment properness to enable participation in various daily life occupations. The sub-scale contains 8 statements rated on a 4-point scale (1=very problematic, 4=excellent). The OSA questionnaire as a whole has good psychometric properties, while the environment sub-scale has established construct validity.

### 2.4 Procedures

The study was approved by the Helsinki Ethic Committee of the Mental Health Center affiliated with the Ministry of Health (approval number 5-21, 7.2.2021). All the participants provided written informed consent following an explanation of the study’s aims and procedures. The PTSD group was recruited from ambulatory services (clinics, day hospitalization, and daycare) of the regional Mental Health Center that provides services to an expansive geographic area. The research team approached individuals who met the inclusion and exclusion criteria. Those who agreed to participate in the study and provided the consent were enrolled. The study procedures with the PTSD group consisted of a single 90-minute session, during which evaluations were conducted in a random order. Following the study, HC completed participation evaluation within the research protocol.

### 2.5 Data analysis

The data was analyzed with IBM’s Statistic Package for the Social Sciences (SPSS), version 27. Descriptive statistics were used to characterize the study participants. The type of distribution was approved using the Kolmogorov-Smirnov test. To examine differences between the groups for demographic variables and participation indices, the Mann-Whitney test, t-test, or χ^2^ tests were used, depending on the type of scale and the type of distribution. In addition, due to the small sample size, we used effect size metrics―Cohen’s d. To test relationships between the study variables in the PTSD group, Pearson’s correlation test was used for quantitative variables with normal distribution and Spearman’s test for ordinal variables or quantitative variables with distribution different from normal. The level of significance in this study was set at 0.05.

## 3 Results

### 3.1 Descriptive statistics of the participants and main variables

The PTSD group comprised 31 individuals, both male and female (women: n=24, 77.4%), with ages ranging from 21 to 53 (M=34.25, SD=9.19). A significant portion of the participants had experienced violent childhood trauma, received a formal diagnosis from 0.5-up to 26.5 years ago (M=5.5, SD=5.7), taking medication, and was officially recognized by the National Insurance as disabled. The majority of participants in this group were single, had more than 12 years of education, held a profession, but had not been employed in the six months before the study, and predominantly resided with their families (**Table 1**). Nearly half of the participants were diagnosed with additional conditions such as personality disorders, depression, and/or anxiety disorders alongside PTSD and had a history of psychiatric hospitalization (**Table 1**).

**Table 1:** Demographic characteristics of the PTSD group (N=31).

		n	%
Family status	Single Married Divorced	18 6 7	58.1 19.4 22.6
Living situation	Alone With roommates Extended family Own family	4 3 15 9	12.9 9.67 48.38 29
Profession	Blue collar White collar No profession	13 3 15	41.93 9.67 48.38
Work in the previous half-year	Yes No	13 18	41.9 58.1
Social benefits	Yes No	27 4	87.1 12.9
Rehabilitation services	Yes No	17 14	54.8 45.2
Age of trauma	Childhood Adult	26 5	83.87 16.12
Secondary psychiatric diagnosis	Yes No	16 15	51.61 48.38
Previous psychiatric hospitalization	Yes No	15 16	48.4 51.6
Neuroleptic medication	Yes No	27 4	87.1 12.9

The HC group included 30 participants, healthy to their report (without diagnoses of any mental or physical disorders), matched by age (M=33.57, SD=8.9; t(58)=0.73, p=0.942, Cohen’s d=0.01) and gender (women: n=22, 73.3%; χ2(1)=6.53, p=0.011) for the study group. However, a significant difference was found between the groups in education level was found between the PTSD group (M=13.47, SD=2.59) and the HC (M=15.4, SD=2.79) (t(58)=-2.774, p=0.007, Cohen’s d=0.71).

### 3.2 Participation: between groups differences

**Figure 1:**
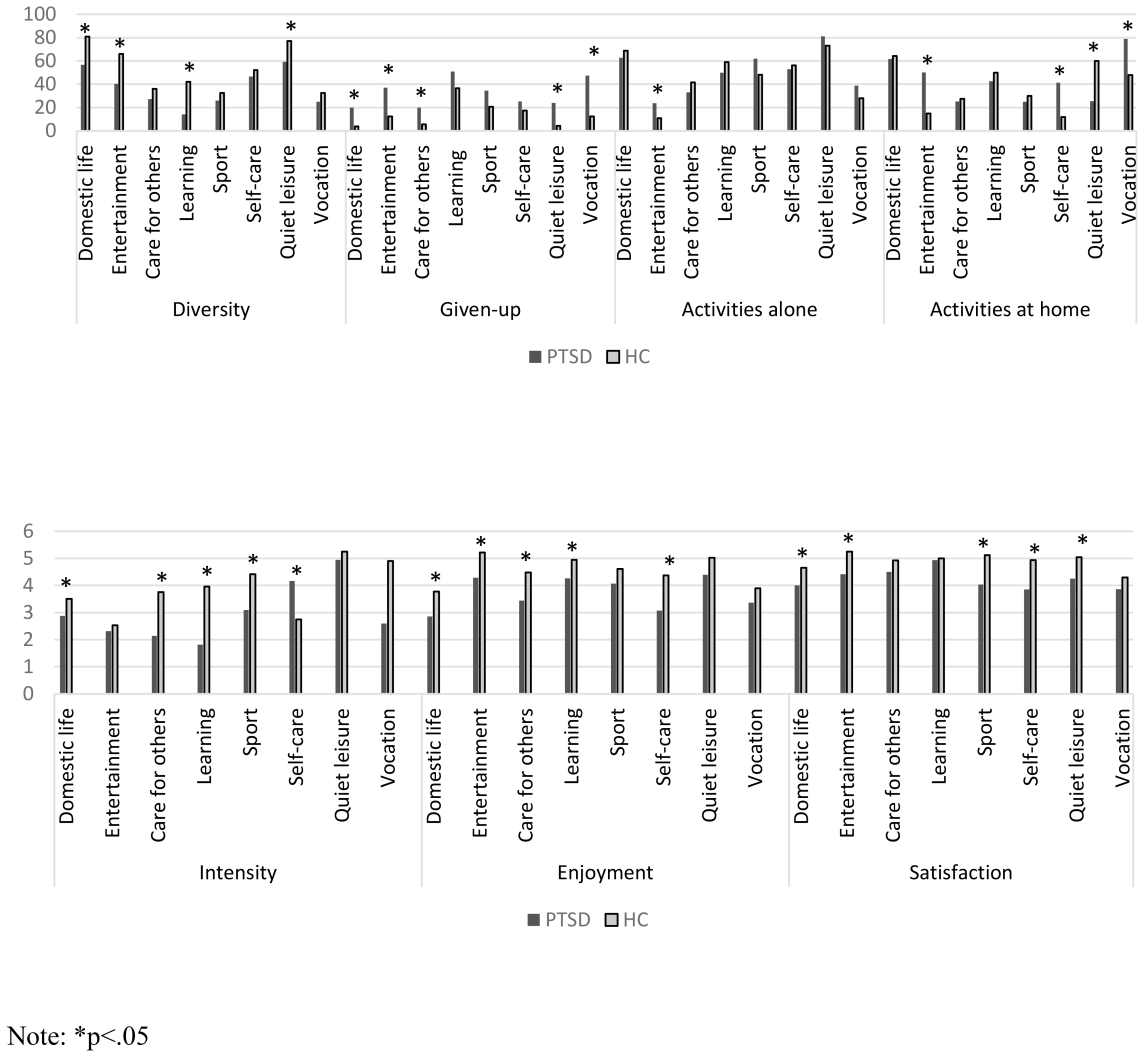
Objective and subjective participation dimensions by groups: PTSD (N=31) and healthy controls (N=30). *p<.05.

A significant difference was found between the groups in the participation intensity (t(58)=-2.42, p=0.018, Cohen’s d=0.62) in favor of healthy subjects (PTSD: M=2.72, SD =0.86; HC: M=3.19, SD=0.62). However, the comparison by areas of occupation revealed that individuals with PTSD participated with a higher frequency in self-care activities (**Figure 1**). A significant difference was also found in the participation diversity (t(58)=-4.73, p=0.000, Cohen’s d=1.22), where HC participated in a wider range of occupations (M=53.4, SD=11) than individuals with PTSD (M=39.23, SD=12.17). In addition, a significant difference was found in the percentage of activities that were given up (Z(128)=-4.76, p=0.000, Cohen’s d=1.4), so that participants with PTSD gave up a higher percentage of activities (M=41.23, SD=12.37) compared to HC (M=19.77, SD=5.93). No significant difference was found between the groups in the percentage of activities performed alone (Z=-1.86, p=0.06; Cohen’s d=0.46), and at home (*t*(29)=-.033, *p*=0.97, Cohen’s d=0.38), and no differences were found in the subjective dimensions of enjoyment (t(58)=-1.61, p=0.11, Cohen’s d=0.41) and satisfaction (total score) (t(58)=-7.47, p=0.46, Cohen’s d=0.19). However, by-area comparison reveals significant differences in enjoyment and satisfaction between the groups in most areas of occupation in favor of HC (**Figure 1**). In addition, the primary location for carrying out occupations varied among the groups by area (**Figure 1**).

A level of assistance in participation and meaning was measured in the PTSD group only. The median level of assistance required to participate was found to be 2.66 (partial help/no-help) (interquartile range 2.38–2.9; the lowest level of assistance is 3 - no help). The mean experience of meaning in occupations was found to be low (M=338.1; SD=195.34; the maximum score is 1560).

### 3.3 Participation dimensions: correlation with personal and environmental factors

The correlation between participation dimensions and personal and environmental factors was investigated in the group of participants with PTSD. A negative correlation of moderate strength was found between the DEX-S and the participation intensity and diversity. Individuals with a higher level of difficulty in executive functioning reported lower participation intensity and diversity. A moderately strong negative correlation was found between the TMT-A score and enjoyment (see **Table 2**). Individuals with higher speed of visual processing reported higher enjoyment of participation. In addition, a strong positive correlation was found between the OSA score and participation diversity, and a moderate negative correlation between the OSA score and the percentage of activities that were given up. That is, individuals whose environment better matched their needs participated with greater diversity and relinquished a lower percentage of activities (see **Table 2**).

**Table 2:** Correlational analysis PTSD group: participation, personal factors and environment (N=31).

	Descriptives M(SD)/Med(Range)	The Adults Subjective Assessment of Participation							
		Intensity	Diversity	Give-up activities	Activities Alone	Activities at home	Enjoyment	Satisfaction	Meaning
MoCA	27 (24-28)	-0.02	0.14	-0.15	0.32	0.18	0.07	-0.11	-0.11
TMTa	34.46 (10.82)	0-.14	-0.31	0.16	-0.09	-0.02	-0.40*	-0.25	-0.28
TMTb	70 (54-110)	.090	-0.27	0.16	0.06	0.06	0.15	0.35	0.03
DEX-S	33.24 (10.68)	-0.45*	-0.46*	0.20	0.31	0.05	-0.23	-0.22	-0.34
UPSA	50.44 (8.62)	.030	0.02	0.23	0.28	0.04	0.03	-0.24	-0.05
PCL-5	52.86 (10.6)	-0.09	-0.23	0.18	-0.21	0.14	-0.35	-0.30	-0.19
GSES	3.09 (0.88)	.270	0.16	-0.07	0.14	0.07	0.30	0.12	0.24
OSA	2.65 (0.56)	.100	.50^**^0	-0.44^*^	-0.30	-0.20	0.16	0.23	0.31
PSQI	6.67 (3.38)	.100	-0.07	0.17	0.47	0.25	0.03	0.15	0.05

*p<.05, **p<.01; DEX-S, Dysexecutive Questionnaire Self reporting; GSES, General Self-Efficacy Scale; MoCA, Montreal Cognitive Assessment; OSA, The Occupational Self-Assessment; PCL-5, PTSD Checklist for DSM-5; PSQI, Pittsburgh Sleep Quality Index; TMT, Trail Making Test A & B; UPSA, USCD Performance-based Skills Assessment.

Significant differences were found in the participation intensity (t(27)=2.609, p=0.015, Cohen’s d=1.06), the participation diversity (t(27)=2.517, p=0.018, Cohen’s d=1.1) and meaning (t(27) =2.813, p=0.000, Cohen’s d=1.3) between individuals with typical (N=7, 23.3%) and atypical sensory avoidance (N=23, 76.7%), so that the last participated with a lower intensity (M=2.49, SD=0.75), a lower variety of activities (M=36.88, SD=11.12), and experienced less meaning in the activities (M=293.09, SD=183.41), in comparison to typical sensory avoidance (intensity: M=3.39, SD=0.92; Diversity: M=48.9, SD=10.52; Meaning: M=506.71, SD=141.72). In the pattern of sensory sensitivity, no statistically significant difference was found in participation indices between subjects with typical (N=4, 13.3%) and atypical (N=26, 86.7%) sensory processing, possibly due to the presence of only four subjects with a score similar to the average. However, a tendency to a difference according to effect size was found in measures of meaning (Cohen’s d=0.66), diversity (Cohen’s d=0.68), and activities that were given up (Cohen’s d=0.34). This finding may indicate that subjects with atypical sensory sensitivity may participate in a narrower variety, give up a higher percentage of activities as well as experience less meaning in occupations. In the sensory seeking scale, a significant difference was found in the percentage of activities performed alone (t(27)=-2.12, p=0.043, Cohen’s d=0.93). Subjects with atypical sensory seeking participate in more activities alone (M=61.41, SD=16.9) compared to those with typical patterns (M=47.4, SD=12.62). No difference was found in the participation patterns between subjects with a typical and atypical low registration index (-1.0<t<1.08, p>0.05).

Correlational analysis examining the association between personal factors, demographics, and PTSD-related data is presented in **Table 3**. It was found that higher age was associated with lower general cognitive functioning. All cognitive performance-based tests and the functional capacity measure demonstrated convergence: higher performance in one test indicated higher performance in the others. Better results in the MoCA and the TMTb tests were associated with perception of the environment as more enabling. Finally, a higher level of PTSD symptoms was associated with lower self-efficacy.

**Table 3:** Correlational analysis PTSD group: personal factors, environment and demographic factors (N=31).

	Age	Education	Illness Duration	MoCA	TMTa	TMTb	GSES	DEX-S	PCL-5	OSA	UPSA
Age											
Education	0.05										
Illness duration	0.3	-0.138									
MoCA	-.355^*^	0.115	-0.275								
TMTa	0.305	-0.046	0.157	-.455^*^							
TMTb	0.281	-0.147	0.212	-.604^**^	.582^**^						
GSES	-0.049	-0.121	-0.201	-0.085	-0.182	0.164					
DEX-S	-0.155	-0.208	-0.004	0.055	0.338	0.269	-0.268				
PCL-5	-0.118	0.054	-0.161	-0.040	0.187	-0.013	-.446^*^	0.268			
OSA	-0.237	-0.044	-0.114	.441^*^	-0.273	-.438^*^	-0.005	-0.083	-0.044		
UPSA	-0.221	0.174	0.025	.539^**^	-.388^*^	-.373^*^	0.076	-0.256	-0.075	-0.018	

*p<.05, **p<.01; DEX-S, Dysexecutive Questionnaire Self reporting; GSES, General Self-Efficacy Scale; MoCA, Montreal Cognitive Assessment; OSA, The Occupational Self-Assessment; PCL-5, PTSD Checklist for DSM-5; TMT, Trail Making Test A & B; UPSA, USCD Performance-based Skills Assessment.

## 4 Discussion

Participation in personally and community-meaningful occupations is an important element of health and well-being (1, 22). As such, the objectives of this pilot study encompassed the characterization of both objective and subjective participation dimensions in individuals with PTSD, as well as the exploration of the relationships between participation patterns and personal and environmental factors. The findings reveal a profound reduction in the objective participation dimensions of intensity, diversity, and the increase in abandonment of activities within the scope of occupation areas. Additionally, there is inferior enjoyment and satisfaction across a considerable number of occupation areas, which is further debilitating. Specific areas of occupation appear to be particularly susceptible to these reductions. The study disclosed factors related to objective participation dimensions, but not to subjective ones; findings that will be further discussed in light of theories and research in the field.

### 4.1 Participation in PTSD

The novelty of this study is a comprehensive delineation of the participation patterns in a range of occupation areas. In line with previous findings on general functional indices (7, 8), an in-depth investigation of participation demonstrates that at a glance, people with PTSD gave up a higher number of activities than healthy controls, and participated in a limited variety of occupations with decreased intensity. Further analysis by occupation areas reveals a pronounced reduction in participation diversity in domestic life activities, leisure activities of entertainment, learning and applying knowledge activities, and, even, quiet leisure activities. In this way, the findings further support the existing literature on specific areas’ limitations (8, 10). But even more important, the study demonstrates that the reduction in diversity occurs horizontally in many areas of occupation, rather than selectively in one area at the expense of others, as may be assumed based on previous research on discrete areas of occupation, such as employment. The vulnerability of the participation patterns in PTSD is even more profound, given the findings on lower general frequency in participation, stemming partially from the same areas with reduced diversity, such as domestic life activities and learning and applying knowledge, but also encompassing additional areas of occupation of taking care of children and others, and leisure physical activities and sport. These findings further expand understanding of the extant participation alterations in PTSD, refuting the assumption that the participation diversity limitations are compensated with the frequency of participation, i.e. people do fewer activities but with higher frequency.

Despite the general reduction in the participation patterns, the study disclosed the complex picture of the participation of those with PTSD arguing for additional investigation. For example, the frequency of taking care of one’s own health was found to be higher in those with PTSD than in the healthy control, reflecting the unique patterns of this population. In addition, even though in general, the locus of participation of individuals with PTSD was similar to healthy, by-area analysis reveals inversion in prevalent places for carrying out activities. Unlike healthy participants, individuals with PTSD engaged in significantly higher percent of entertainment, vocational, and self-care activities at home, while less of their quiet leisure activities occurred at home. Different environments can reflect the choice or lack of choice of the people. Still, in both cases, it determines different supports, opportunities, and requirements, intervening substantially with the participation. The participation patterns in PTSD raise even more concern given the findings on greater abandonment of occupations, suggesting that the limitations may be progressive. It is important to note, that occupations were waivered in areas that have been found to be deficient based on other indices: domestic life, leisure activities of entertainment, taking care of children and others, and quiet leisure activities, delineating the particularly susceptible areas of occupations.

Unexpectedly, the summary score of subjective participation dimensions of enjoyment and satisfaction was found to be similar between healthy individuals and those with PTSD. The results may indicate that actual participation in occupations can serve as a preventive factor against alterations in participation experience. Still, the areas analysis reveals that both enjoyment from the participation and satisfaction with it was inferior for individuals with PTSD in a range of investigated areas, including domestic life, self-care, and leisure activities of entertainment. In addition, we found lower satisfaction with sports and quiet leisure activities and lower enjoyment from participation in occupations of caring for children and others, and learning. These findings may represent the impact of core trauma mechanisms on participation as they are obstructive to positive emotions and cognitive appraisal (DSM-5) (2). Or, in the case of satisfaction, the results may reflect the dispersion between actual participation and capacity and/or expectations.

Overall, the findings indicate that PTSD posed restrictions on most objective and subjective participation dimensions in both more obligatory and structured occupations (e.g., domestic life activities) and occupations of personal choice and preference, such as leisure. The importance of this understating should be discussed in light of the role of these two clusters of occupations in life and efforts to detect possible pathways for advancing participation. Although there are certain differences in their roles, both clusters of occupations enhance confidence, self-esteem, and sense of control, and provide a platform for skill development and maintenance, including coping with stress and disability (15, 60). Domestic life activities enable independence in everyday life, ensure satisfaction of basic needs, have standards for performance, supporting instrumentally participation in additional areas (15). For example, independence in transportation is an important enabling factor for employment, leisure, and social participation. Whereas practicing leisure activities brings unique meaning that cannot be obtained through other occupations: it enables a sense of freedom and provides opportunities for self-expression, social engagement, and connection, and for physical and mental relaxation from obligations and routines (60). Thus, limitations in both clusters of occupations place individuals with PTSD in an unfavorable position as to their health and well-being. Next, based on features of these occupations, it is less likely to be assumed that general participation promotes strategies such as occupational structuring (e.g., through rehabilitation services) or enables unlimited access to a wide range of occupations (e.g., through social welfare programs), resulting in a breakthrough and fostering participation among this population. Still, there are encouraging findings, since no differences were found between the extent of participation with others versus alone, implying that drawbacks of loneliness in participation may be less prominent in PTSD.

### 4.2 Enablers for participation

Our findings contribute to disclosing possible mechanisms facilitating objective and subjective participation dimensions. First, we found that both diversity and intensity of participation were associated with individual appraisal of executive and emotional dysregulation in daily life situations. The study expands previous literature, approving the association between self-perception of skills and comprehensive measurement of participation, over general functional indices or disability scores, and demonstrating this phenomenon for individuals with cognition and functional capacity within normal range. These, while previous studies involve people with impaired cognition (39, 42, 61). In discrepancy with some previous studies (39), objective participation dimensions were unrelated to cognitive performance as well as functional capacity. The findings imply the importance of self-perception of skills and competencies for participation, rather than their actual level in PTSD. Or, it may be suggested that self-reporting may be more sensitive than objective tests to the change that occurs following trauma. Moreover, we demonstrated that functioning-specific self-perception was of importance for participation, rather than the estimation of general self-efficacy which was found to be detached from the participation. Still, caution is needed since the objective participation was measured in this study based on self-report, thus it might be assumed that the findings replicate previous studies in PTSD on convergency between self-reported tools, but not with performance-based ones (23, 42, 61).

Next, we found sensory modulation alterations of all types of combinations between the sensory threshold (low versus high) and behavioral patterns (active versus passive) in our cohort, indicating a prevalent pattern of passive behavior either for a low or high sensory threshold or active behavioral avoidance in the case of low threshold. These findings further expand previous literature on SMD in PTSD (45). Innovatively, this study found an association between sensory alterations and participation limitations, disclosing distinctive characteristics of the population with PTSD; as in other serious mental diagnoses, this association has been hardly found (43, 44). We first provide research evidence for intuitive assumption in PTSD demonstrating a link between active avoiding behavioral response due to low sensory threshold―sensory avoidance, lower diversity, and intensity of participation in daily life. We also reveal that individuals who minimize their behaviors, even though there are high sensory thresholds, find themselves participating more alone, and individuals with sensory sensitivity, which represents passive behavioral strategies to a low sensory threshold, waiver on more occupations and participate with inferior diversity. These findings disclose an additional role of sensory disorder in PTSD, being not only an integrated part of symptomology (45) but also a hindering factor for participation and reintegration in daily life.

Human and non-human environments were found to be supportive factors for the objective participation in PTSD. The environment had been addressed in PTSD mostly through the lens of the source of trauma, symptomology, and interpersonal relationships (62). Environment provides instrumental means for participation in occupations of self-care, productivity, leisure, and social context through attitudes toward occupation and emotional support (1, 63). Extending previous research on various health conditions (63, 64), our findings suggest the critical role environment plays in PTSD. This impact may be beyond personal factors, but such an assumption should be further approved by the research.

Interestingly, in discrepancy with the previous studies (24–27), we found that the severity of PTSD symptoms and sleep disturbances were not associated with objective participation dimensions, even though the symptom levels were quite high in the study’s population (51). This significant disparity may stem, again, from the difference in the participation evaluation (comprehensive in-depth evaluation versus brief general index (23, 27) and study population: those exposed to trauma (e.g., 27) versus those with a formal diagnosis. These findings suggest that the symptoms’ alleviation may not enhance participation in a wide range of life areas for those who developed PTSD with moderate symptom severity.

Additional surprising findings are that neither symptom severity nor self-efficacy and self-reported measurement of executive and emotional dysregulation were associated with subjective dimensions of participation satisfaction, enjoyment, or meaning. Only the measurement of complex attention and speed of processing was found to be associated with enjoyment. We assume that these findings reflect the quality of fulfillment of the questionnaire, rather than indicate a credible phenomenon. Indeed, we found that performance-based measures (i.e., cognitive and functional capacity) show convergence, whereas most of the results from self-reported tools were not significantly related to each other. Given the vulnerability of the subjective dimension in PTSD that was found and its importance for health and well-being, the results raise a concern. Further research is needed to advance the modeling of subjective participation dimensions since current theoretical and research insights on factors intervening with these dimensions in PTSD are deficient.

### 4.3 Limitations

There are several limitations in this study. The study groups were matched by age and gender; however, they were found to be different for the level of education. Since education may affect participation, we recommend addressing it in future studies. In addition, the control group was recruited based on self-report on intact health, while no cognitive and other tests for health-related conditions were managed. It is recommended that in future studies, health status be confirmed through medical charts and health-related measures, including measures similar to those of the study group, be included in the study procedures to ensure the eligibility of participants for the control condition. Next, considering the limited convergence between self-reported tools, as well as between self-reported and performance-based measures, we recommend further investigation into the interplay between different constructs in PTSD and the impact of different measurement approaches. Still, the most deliberating limitation is a relatively small number of participants in the PTSD group. Given numerous comparisons that were done in the study, the study enables indication of trends rather than well-established conclusions. Additionally, the small number of participants with PTSD might be of particular effect for specific analysis, such as in the case of SMD, while the differences were investigated within the PTSD group only. Another issue that may have an impact on the generalization of the study’s findings is diagnostic criteria since individuals with several sources of trauma within different timelines were enrolled in the study.

### 4.4 Conclusions

To summarize, individuals with PTSD have unique participation patterns and unique enablers for participation. Participation limitations followed coping with PTSD are inclusive, involving objective and subjective dimensions, of both obligatory and non-obligatory occupations. This situation is of particular concern regarding health, well-being, and recovery opportunities following PTSD. Given the extensive participation restrictions, it may be assumed that general rehabilitation and intervention strategies for the participation promotion may be less effective for populations with PTSD. Therefore, intervention approaches, dedicated to address unique challenges of this population, are required. Based on the results of this study, it is recommended to consider addressing individual appraisal of cognitive functioning, sensory modulation, and environmental factors as facilitators for objective participation dimensions, rather than focusing solely on the severity of PTSD symptoms, objective cognitive performance, or functional capacity. Additionally, it should be noted that subjective dimensions of participation remain largely unexplained by the study’s variables and are poorly understood, which limits interventions aimed at promoting these dimensions. Given the nature of this pilot study, further large-scale research is needed on participation in PTSD to alleviate its impact on health and well-being.

## Data availability statement

The raw data supporting the conclusions of this article will be made available by the authors, without undue reservation.

## Ethics statement

The studies involving humans were approved by The Jerusalem Mental Health Center, The Ministry of Health, Israel. The studies were conducted in accordance with the local legislation and institutional requirements. The participants provided their written informed consent to participate in this study.

## Author contributions

RS: Formal analysis, Investigation, Writing – original draft. YBG: Data curation, Investigation, Writing – review & editing. LL-V: Conceptualization, Formal analysis, Methodology, Supervision, Writing – review & editing.
